# A qualitative exploration of physical, mental and ocular fatigue in patients with primary Sjögren's Syndrome

**DOI:** 10.1371/journal.pone.0187272

**Published:** 2017-10-31

**Authors:** Rebecca J. Stack, Sue Southworth, Benjamin A. Fisher, Francesca Barone, Christopher D. Buckley, Saaeha Rauz, Simon J. Bowman

**Affiliations:** 1 Centre for Translational Inflammation Research, University of Birmingham, Birmingham, United Kingdom; 2 Academic Unit of Ophthalmology, Birmingham and Midland Eye Centre, Birmingham, United Kingdom; 3 Department of Rheumatology, University Hospitals Birmingham NHS Foundation Trust, Birmingham, United Kingdom; National Institute of Dental and Craniofacial Research, UNITED STATES

## Abstract

**Introduction:**

Primary Sjögren’s Syndrome (pSS) affects exocrine glands such as those producing the tear film, leading to dry and painful eyes, but is also associated with fatigue. The experience of fatigue in pSS, and its relationship with sicca symptoms, is poorly understood.

**Methods:**

Twenty people diagnosed with pSS were recruited to participate in a semi-structured qualitative interview about their symptoms experience. Interviews were audio-recorded, transcribed verbatim and analysed using thematic analysis.

**Results:**

People with pSS described physical tiredness, mental fatigue and ocular fatigue. Mental fatigue was characterised by difficulties in attention, particularly, the ability to follow conversations and short-term memory problems. Participants linked their experience of fatigue to feeling of depression, frustration, irritation and anxiety, and therefore, fatigue was suggested to have had a large impact on their psychological well-being. People with pSS also described a range of ocular symptoms including pain, dryness, and itching, which were compounded by fatigue. For some, eye fatigue was pervasive, and daily activities involving the eyes such as reading, using the computer and driving were impaired. In some cases, the level of ocular discomfort was so severe it prevented sleep, which in turn impacted on general fatigue levels.

**Conclusions:**

People with pSS experience fatigue in a range of ways; physical, mental and ocular fatigue were described. Fatigue was suggested to exacerbate other ocular symptoms, posed serious physical limitations and caused psychological distress. Further research into the nature of fatigue and ocular symptoms in pSS is required.

## Introduction

Primary Sjögren’s syndrome (pSS) is an autoimmune condition that results in inflammation of exocrine glands, particularly the lacrimal and salivary glands. As a result tear and saliva production is reduced, causing patients to experience ocular and oral dryness. However, similar to other rheumatic conditions, pSS is also characterised by fatigue [1] and sleep disturbance.[2] Fatigue is a severe problem experienced by many patients diagnosed with rheumatological and musculoskeletal conditions. Approximately 70% of patients with pSS suffer from disabling levels of fatigue.[3] Fatigue has been found to have a negative impact on health related quality of life and has associated with poor physical functioning and general health.[4] Studies have found high levels of fatigue in patients with pSS, in a study of 94 patients, 67% were found to experience clinical levels of fatigue with somatic fatigue being more prevalent (somatic and mental fatigue was experienced in 96% and 48% of patients with pSS respectively).[5]

Fatigue has major consequences for patients’ lives. Previous qualitative explorations of Sjögren’s Syndrome have attempted to characterise the range of symptoms experienced by patients, in which weariness and fatigue were major symptoms.[6;7] In a qualitative exploration of fatigue in pSS, Mengshoel and colleagues, reported sensations of bodily heaviness and how patients had reduced their involvement in daily activities to cope with fatigue. In addition, the uncertainty and unpredictability of fatigue in pSS was highlighted.[8] Such qualitative explorations are vital for understanding patients’ lived experiences of fatigue and its impact on patients’ activities, and ability to cope. However, research in RA suggests that fatigue is multi-faceted, and interacts with the experience of other symptoms. Further explorations of fatigue in pSS is required and should take an inductive approach to the exploration of symptoms and their interaction with commonly presenting sicca symptoms in pSS.

Barendregt and colleagues highlighted that studies characterising fatigue in pSS are rare.[9] Furthermore, many studies have highlighted that fatigue is often under-recognised in rheumatology settings, and not addressed clinically.[10;11] In part, this may be due to a poor understanding of the complexity of fatigue and the different symptom presentations patients with pSS may display related to fatigue. The aim of this exploratory study was to investigate the experience of fatigue and related symptoms in patients with pSS.

## Methods

Ethical approval was obtained from HumberBridge Research Ethics Committee. All participants provided informed written consent.

### Interview participants

Participants were adults (aged > 18) with a physician confirmed diagnosed of pSS. Participants were recruited through the Optimising Assessment of Sjögren's Syndrome (OASIS) observational study with the support of the Birmingham National Institute for Health Research Wellcome Trust Clinical Research Facility. In the OASIS cohort, the mean EULAR Sjögren's Syndrome Patient Reported Index (ESSPRI) score in patients with pSS is 6.83 (SD 2.06; n = 63). The ESSPRI is a widely accepted patient reported outcome calculated from the mean of three 0–10 scales for dryness, fatigue and pain [12]. The patient acceptable symptom state is <5.[13] In the OASIS cohort the score for the ESSPRI fatigue domain was 7.08 (SD 2.37) indicating high levels of fatigue. Importantly the fatigue domain of the ESSPRI correlates very strongly with the respective domain of the more complex Profile of Fatigue and Discomfort-Sicca Symptoms Inventory.[14] A self-selecting sample of 20 people with pSS from the first 63 participants recruited to the OASIS cohort volunteered to be interviewed.

### Interview procedure

Interviews were conducted by one observer (RS), and were semi-structured guided by an interview schedule. The initial structure of the interview schedule was developed in collaboration with patient research partners diagnosed with pSS, who provided insight into the lived experience of pSS, and how participants may respond to the themes and questions covered within the interview schedule. In addition, patient research partners revised the initial interview schedule structure and modified the interview questions according to their personal experiences as individuals with a lived experience of pSS. The interview schedule primarily consisted of open ended questions to elicit patients’ experience of symptoms, coping strategies and methods of self-management of pSS. The initial interview schedule was changed inductively to reflect and incorporate the topics discussed by early interview participants.

The interviews were conducted in a hospital out-patient department, or research facility. Each interview lasted between 30 and 90 minutes. All interviews were audio recorded and transcribed verbatim. Data were collected until saturation had been reached with no new themes emerging.

Data were analysed using thematic analysis.[15] Data collection, transcribing and analysis of interviews were undertaken in parallel to ensure that the themes derived from earlier interviews could be incorporated into later interviews. Initial line-by-line coding of the interview transcripts was undertaken by RS. Line-by-line coding allowed analytical summaries of each interviewee’s accounts to be produced. Independent blind coding was undertaken with two Patient Research Partners on two interview transcripts. Following validation, the initial codes were grouped together into the most noteworthy and frequently occurring categories and core themes were developed. The data presented in this paper focuses on participants accounts of fatigue, and coping with fatigue.

## Results

A total of twenty people diagnosed with pSS were interviewed. The participants’ ages ranged from 19–90 years (mean 54 years of age). All participants were female. Illustrative quotes can be found in Tables 1–3. The quotations presented in these tables are drawn from a larger pool of quotations on symptom management which can be viewed in an online supplementary table.

**Table 1:** Quotations related to theme 1: The physical experience of fatigue.

Quote no.	Participant no.	Quotations
1	15	*“I come out of that horrible “can’t get off the couch” feeling*, *because I could be on the couch for two days*. *Just have to sleep and rest and just can’t do anything*.*”*
2	3	*“You can feel reasonably normal and then all of a sudden it's just like an overpowering shatterness*, *you just can't stay awake*, *I've sat down here and I've just slept for two or three hours or maybe an hour here and then an hour again in the evening and then gone to bed*.*”*
3	1	*“The fatigue*, *all of a sudden you can be out shopping and you think*, *‘Oh*, *my God*, *I’ve got to go and sit down*.*”*
4	10	*“Absolutely exhausted before I even start in the morning*, *because I feel tired anyway when I wake up in the morning*, *so I try not to overload in that way ……I am still absolutely exhausted in the afternoons and the early evening…I’m absolutely bushed (exhausted) and literally struggling at that point then*, *I feel like my body just doesn’t want to move anymore*.*”*
5	15	*“Because you don’t want to but you just have to think ‘well*, *I just need to rest now’…you just have to give in to it really*. *Fatigue is very bad I’m just finding I’m on the couch sleeping regularly*, *I suffer from tiredness anyway*, *particularly low at the minute”*
6	14	*“I battle fatigue every day*, *but I don’t let anybody see it*, *but the fatigue is horrendous*. *Sometimes I could fall to sleep standing up*. *And I think I disguise it quite well*, *to be perfectly truthful*. *People only see what I want them to see*. *They don’t see me going back to bed in the morning*, *they don’t see me trying to get out of bed at eight and can't get up*, *you know*? *They don’t see me falling to sleep and I cover everything up*.
7	2	*“Sometimes I’m embarrassed*. *I’m embarrassed because I can’t do as much as I want to do or used to be able to do*.*”*
8	15	*“I’m linking it up to low feelings*. *I’m just feeling dreadful*. *I feel like I’m walking through treacle*, *everything I do*, *even up to the simplest unloading the dishwasher can feel like climbing a mountain*. *It’s just ridiculous minor exertions can feel huge and I feel breathless*. *It’s quite hard work for people that haven’t got much energy and are a bit low*.
9	10	*“Especially when you’re tired*, *you feel lower*. *If I’m tired and something bothers me*, *I’m more likely to feel*, *well I say cry*, *but I can’t cry but you know what I’m saying*, *but for like*, *breaking down and saying I can’t do it*, *when you’re tired*.*”*

**Table 2:** Quotations related to theme 2: Ocular fatigue and fatigue related to ocular complaints.

Quote no.	Participant no.	*Quotations*
1	*13*	*“Where I work I'm looking at a computer all day and I find that they get very*, *very tired and sore by the end of that*. *Then I'm unlikely to come home and start reading much because they're sore and tired*. *They do get a bit blurry*.*”*
2	*5*	*“I used to be able to cope with loads of stuff*, *especially in the evenings*. *That was the time when I was most productive*. *Now I can't because either I'm tired or my eyes can't cope*, *because by the evening the eye that's the worst gets really gritty*, *however many drops I put in it*. *And my evenings are being impeded*. *I used to do a lot on the computer in the evening and that's not good either”*.
3	*20*	*“I can’t read in an evening*, *for example*, *because they're just so sore and tired*. *I certainly don't drive any more in the evening because I don't feel confident that my eyes—that I'm seeing clearly*.*”*
4	*11*	*“It’s not so much that they [eyes] are tired*, *they are just very very dry*. *The itchiness can prevent me sleeping at nights*. *I usually get 4 or 5 hours*, *sometimes I don’t sleep at all*. *If I get itchiness I don’t sleep*. *It’s the itchiness that prevents me from sleeping*.*”*
5	*11*	*“I read*, *and I have a lot of problems lately*, *I find that I can’t read very well*. *I can’t seem to see the writing*, *and my eye sight isn’t very good now*. *I have to write quite big*, *with my writing*. *When I’m reading my eyes do hurt me*, *it takes me a long time to read because I can’t see very well and it’s hurting*, *and I can’t ready very well*, *I get tired*. *I can’t watch the TV too long*, *I have to have a rest*.*”*
6	*10*	*“My eyes*, *trying to keep my eyes open*, *you just feel that exhausted*, *that you just can’t seem to get yourself the ‘umph’ to even going upstairs to go to bed*, *it’s just ‘oh God*, *I’ve got to go up’*. *It [fatigue] can happen at any point because I feel tired a lot*, *you just*, *they [eyes] just feel very heavy*, *very gritty and it’s just*, *you feel like they’re struggling to open*, *I’ll just sleep but it can happen at any point*.*”*
7	*16*	*“I fluctuate*, *I can sleep like a log but I do have to get up to do my eyes every night because they’d be unbearable in the morning if I didn’t*.*”*
8	**3**	*“The eyes are so bad*. *Once I collapsed and broke into tears it made me so stressed*. *I rather put it down to stress and slight depression*, *but my eyes were bad as well*.*”*

**Table 3:** Quotations related to theme 3: Cognitive aspects of fatigue.

Quote no.	Participant no.	*Quotations*
**1**	**11**	*“And sometimes I can’t think*, *and I can’t concentrate*. *Very difficult to concentrate*. *If we want to go somewhere*, *normally we go out dancing*, *and it’s hard to concentrate on the dancing*.*”*
**2**	**17**	*“I do forget*. *I am more forgetful*. *So I have noticed I get tired a lot quicker in conversation*. *I'm tired now*, *and sometimes I'm tired and as soon as I get…it's the fatigue of the illness*.*”*
**3**	**15**	*“I get foggy thinking*, *so I feel like I have a conversation with someone and then I can’t remember what we’ve spoken about particularly I’ll forget the details…*..*like I say short term memory’s not very good either so there’s quite a few things really*.*”*
**4**	**2**	*“But concentration is much more difficult; it was difficult before but it’s even more difficult now*. *Yeah*, *staying focused*, *and short-term memory appalling now*, *appalling [yeah]*, *yeah*, *that I’ve notice*. *It absolutely does my head in*. *When you’re in a conversation*, *if it’s quite a fast conversation*, *I find that difficult to do now*, *whereas before I could just*, *you know*, *do all of that*.*”*
**5**	**19**	*“Sometimes I do struggle to do a word search and sometimes I can't identify things*, *but again*, *it isn't a problem because as long as I can get to the shops*, *get my food*, *I know what I'm eating…*.*Sorry*, *I've forgotten what the question was*? *“*
**6**	**4**	*“So probably…that is quite bad*, *the fatigue*, *really dreadful*, *the fatigue*. *Yes*, *very tiring*. *This will whack me out now*, *this interview*.***“***
**7**	**7**	*“But it was just—I felt as if I was wading through treacle*.*”*
**8**	**15**	*“You feel like you’re working hard to focus on the conversation and I think some of it is you’re distracted by the tiredness and the pain*, *you know I’ve got constant pain so that’s very distracting anyway*. *But some of it is just fogginess in my head and it is difficult because it’s got worse over the years*, *I was pretty sharp and very good at my job when I was working*, *very sort of switched on and I think that’s got worse over the years and the thought of doing something like I used to do now I just don’t know if I could do it really*, *I couldn’t take on a big mental challenge like I used to you know so that’s yeah that’s tricky really*.*”*
**9**	**10**	*“I’ve noticed myself when I’m trying to read small print*, *I’m having to sort of really concentrate on trying to focus and move it about to read it*, *whereas before I never used to have an issue*.*”*
**10**	**11**	*“I feel very very tired*, *at night I can’t sleep very well*. *I feel tried but I can’t sleep*. *I feel tired*, *I feel very very irritable at the moment*. *I was never*, *like that before*, *I’ve become very very bad tempered*. *I keep on having arguments with my husband*, *I’m very impatient*, *and I don’t know if this is due to my illness*, *I know I wasn’t like this before*, *I get anxious*.*”*

### Theme 1: The physical experience of fatigue

Participants described their fatigue as a “*horrible*” and as being a disabling part of their illness which often left them immobile for long periods of time (Table 1, Quote 1, further referred to as T1Q1); some episodes of fatigue resulted in sleeping for days at a time (T1Q1). When episodes of fatigue struck participants spoke about total physical exhaustion, feeling “*shattered*” and their body not wanting to move (T1Q2). In some cases the onset of fatigue was sudden, unexpected and unpredictable, with some participants being concerned that an episode of fatigue could strike at any time during the day (T1Q3). However, others had noted the time of the day when fatigue was mostly to strike and were able map their daily activities around their fatigue to ensure they were able to rest during times when fatigue was likely to peak (T1Q4). Participants also spoke about giving in to the tiredness, and just accepting it by resting (T1Q5).

Participants described the everyday battles associated with fatigue, but were keen to hide their difficulties from others and spoke of covering things up (T1Q6). Some described being embarrassed by tiredness and not wanting others to see the effect their symptoms were having on them (T1Q7). One participant described how the large physical burden left her feeling dreadful, and caused her to feel low (T1Q8 –she also described feeling as though she was walking through treacle (a thick and sticky dark syrup), which is discussed further by other participants in relation to cognitive difficulties under theme 2). Simple tasks had become highly exhausting and were impacting on her psychological well-being. One participant spoke about her tiredness and stress that were sometimes so great that she could be brought to tears (T1Q9). However, due to pSS, the ability to physically produce tears was impaired.

### Theme 2: Ocular fatigue and fatigue related to ocular complaints

Many participants described experiencing feelings of tiredness in their eyes. For many participants there was an interaction between the experience of ocular fatigue and other eye symptoms such as blurred vision (T2Q1), grittiness (T2Q2) soreness (T2Q1,Q3,Q5) dryness and itchiness (T2Q4). In some cases, participants described their eyes as being unable to “cope” regardless of the use of interventions such as drops (T2Q2). Eye related fatigue, at its worst, prevented any daily activities from being undertaken. In some cases there were times of the day when participants were unable to undertaking routine activities which involved using their eyes such as reading during the evening (T2Q1), writing, watching TV (T2Q5) using the computer (T2Q2) or driving (T2Q3).

For some, ocular tiredness was linked to their physical fatigue and feeling exhausted towards the end of the day (T2Q6). One participant described a combination of physical fatigue, gritty eyes and struggling to keep her eyes open. This combination of symptoms was described as being unpredictable and resulted in the need to “just sleep” (T2Q6). Some participants found it difficult to understand whether the tiredness they felt in their eyes was due to general tiredness, or a fatigue specific to the eyes. In some cases, the level of discomfort in the eyes was so severe it prevented sleep, which had, in turn, an impact on general fatigue levels (T2Q4). Some participants described how ocular symptoms were interfering with sleep, which was a contributing factor to their overall tiredness. In one case tiredness in the eyes was linked to the presence of other ocular symptoms at night which included pain and grittiness (T2Q4). Others found that they needed to get up regularly during the night to attend to their eyes, either to address immediate discomfort, or to prevent extreme discomfort in the morning (T2Q7). Finally, like physical fatigue, eye symptoms and fatigue caused stress and feelings of depression in one participant, who described collapsing in tears (T2Q8).

### Theme 3: Cognitive aspects of fatigue

Difficulties in concentration prevented some from undertaking daily activities (T3Q1). Holding a conversation was described as a difficult task by many participants, as they were finding that they became more tired as the conversation went on and their concentration abilities became exhausted (T3Q2). Participants also described being forgetful (T3Q2), but also described forgetting words or concepts mid-conversation, with one participant describing how during conversations she is unable to recall details of the current conversation (T3Q3). Fast conversations were described as particularly difficult to follow, and participants noted a decline in their ability to engage with conversations since being diagnosed with pSS (T3Q4). Multiple participants described short-term memory difficulties (T3Q3&Q4). Other participants reported struggling for words, and being unable to identify familiar objects (T3Q5). Interestingly, for some participants’ cognitive fatigue appeared during the interview, and meant that the interview had to be halted or brought to close. The cognitive strain brought about by the interview was apparent when participants described their difficulties in focusing on the questions being asked (T3Q5), and feeling as though their concentration was drifting during the interview. One participant was aware that taking part in the research interview for this study was going to leave her feeling exhausted (T3Q6).

Participant also described feeling generally slowed down, *“fogginess”*, and feeling as if they were *“wading through treacle”*(T3Q7&Q8). One participant distinguished between times when her cognitive difficulties were caused by being distracted by the tiredness and the pain, and other times when her cognitive difficulties were due to “*fogginess*”, and suggested that the latter was getting progressively worse (T3Q8). The worsening of these symptoms meant that she felt less sharp, and was unable to take on mentally challenging tasks. One participant spoke about difficulties in reading and that concentrating on the print was a real difficult for her (T3Q9), this is in contrast to the previous theme where difficulties in reading were associated with soreness in the eyes (T2Q3) and pain (T2Q5).

When describing the impact of fatigue on cognitive function many participants also described how fatigue had caused them to feel low, and in some cases depressed. Participants also described feeling bad tempered and anxious as a result of their fatigue. In some cases the culmination of physical and mental fatigue has led to feeling “low”, with participants describing their attempts to seek help for their feelings (T3Q10). In some cases the physical, cognitive and emotional aspects of fatigue were inseparable, and were clearly interlaced, for example participant 15 described physical fatigue, walking though treacle, and feeling low as being an integrated experience (T1Q9).

## Discussion

People with pSS reported experiencing fatigue in multiple ways. This study highlights the physical and mental components of fatigue, the experience of ocular fatigue, and fatigue exacerbated by the presence of ocular symptoms including irritation, dryness and ocular pain. Other studies in the field of rheumatology have identified different types of fatigue in patients with disease such as RA; making the distinction between severe weariness and dramatic overwhelming fatigue for example.[16] However, this study highlights that people with pSS report multiple components of fatigue which may relate to the systemic nature of pSS and presence of sicca symptoms.

Previous research has found that fatigue plays a major role in the lives of people with pSS, and because of its complex and multifaceted nature, fatigue has an impact on physical function, cognitive abilities, visual functioning and emotions. This study highlights that people with pSS described the interrelated nature of fatigue symptoms. Some participants were unable to identify whether they were unable to read due to a fatigue specific to the eye, or a more general cognitive fatigue. Fatigue also played a major role in participant’s ability to use their eyes, and engage in visual activities such as driving, or using a computer. In addition, sleep was also affected by eye problems experienced at night. Clinically the assessment of fatigue and the impact of fatigue on ocular complaints many need to be enhanced to ensure that the full impact of symptomology is assessed, however, further research would be required to assess whether in-depth assessment would result in enhanced patient outcomes. In addition, quantitative research should be undertaken to examine the number of people who present with particular types of fatigue, or if patterns of symptoms exist. Additional we recognise that potential associations between different patterns of fatigue with distinct clinical profiles (e.g. different phenotypes and ESSDAI scores) need to be explored and reported.

In addition, while some coped in practically orientated and pragmatic ways, other patients were overwhelmed by their symptomology. From participants accounts the emotional impact of fatigue appears to precede feelings of depression and anxiety. This finding indicates that psychological support maybe beneficial for patients who struggle to coping with uncontrolled fatigue and ocular symptoms. Other research already supports the need for psychological support in this population; for example, the prevalence of clinical anxiety and depression is higher in people with pSS when compared to controls.[17–19] Forty-eight percent and 32% of patients with pSS were found to be diagnosed with “possible” clinical levels of depression and anxiety respectively,[20] and this psychological morbidity in pSS is predictive of fatigue levels.[21] In this qualitative study, quantitative measures of fatigue and depression were not administered to this cohort, however, research examining whether depression is predicted by different components of fatigue should be undertaken in a larger cohort of pSS patients. Quantitative research is being undertaken by our team to understand the impact of fatigue on existing levels of psychological morbidity and perceptions of illness.

Participants described the experience of physical and mental fatigue as having a profound impact on psychological well-being. With many people describing the experience of fatigue as severe and life-limiting. The physical experience of fatigue was suggested by participants to lead to episodes of extreme exhaustion, an inability to physically move and the need for sleep for extended periods of time. In some cases the onset of physical tiredness was sudden and unpredictable, leading to uncertainty. Studies have found that mental fatigue is particularly problematic for patients with pSS.[5;9;22] Associations have been found between mental fatigue, hopelessness and depression.[5] It is also possible that patients may have cognitions about fatigue which interact with negative emotions. For example, catastrophising about fatigue may lead to feelings of hopelessness and eventual depression. Wouters and colleagues found that patients with worse fatigue had higher levels of activity avoidance, a greater somatic focus and engaged in less physical activity.[23] This evidence supports the argument that cognitive and behavioural components interact in patients experience of fatigue. However, future research must explore the role that negative cognitive appraisal of physical symptoms has on psychological well-being. However, we must also acknowledge that the physical and psychological manifestations of fatigue may not interact and may actually exist in isolation.

This study highlights the complex relationship between fatigue and sleep in people with PSS, some participants describe fatigue independently of ‘sleepiness’, sleep related problems, however, in other cases sleep problems and fatigue were described in an intertwined manner. Further explorations of the cognitive components of fatigue in pSS may lead to interventions which impact on patient well-being, and may lead to better quality of sleep in affected patients. Participants also described how sleep was problematic, this was due either to eye irritation preventing sleep, or the continued need to apply eye drops during the night. Other experimental studies have shown that fatigue and joint pain increased following poor sleep.[24] This may occur because poor sleep interferes with cognitive processes, and may reduce one’s ability to self-regulate responses to pain, or may interfere with the regulation of neurochemical analgesics.[25] Further research is needed to understand the impact of sleep disturbance (whether due to ocular irritation or pain) in people with PSS.

Limitations of this research were that all participants interviewed were female. However, 90% of all patients diagnosed with pSS are female and as such, this investigation therefore, closely reflects the general pSS patient population. However, like other research in the field of rheumatology, we recognise that male views are under-represented, and advocate further research exploring the experience of men with rheumatic conditions. In addition, the presence of other comorbidities which may have contributed towards fatigue were not controlled for in this sample, future research should consider the role of anaemia, depression and sleep disorders in patients with PSS.

This study, has highlighted the complex nature of fatigue experienced by patients with pSS, and highlighted the importance of considering cognitive fatigue and the interaction of fatigue with ocular symptoms. However, we are limited in our ability to unpick the intricate interactions or causal mechanisms between ocular, mental and physical fatigue. However, longitudinal assessments of patients symptomology may give an indication of how symptoms evolve, and whether early control of ocular symptoms can reduce future occurrences of ocular related fatigue and mental fatigue. Furthermore, the concepts highlighted in this research could contribute towards a more robust clinical assessment of fatigue, ocular symptomology and the impact of these symptoms on daily functioning.

This study highlights the disabling nature of fatigue for many patients with pSS and suggests that more attention should be given to patients experience of fatigue and ocular symptoms. The nature of the relationship between ocular and systemic symptoms and ocular and somatic fatigue has been discussed by patients and merits further exploration.

## Key messages

People with primary Sjögren’s Syndrome (pSS) experience physical, cognitive and ocular forms of fatigue.

Ocular symptoms such as eye pain, soreness and irritation appeared to interrelate with fatigue.

Fatigue had a large impact on routine activities, and was reported to cause anxiety and depression.

The assessment of fatigue and ocular symptoms must be improved.

People with pSS need may psychological support to cope with the emotional impact of symptomology, however, more research is needed.

## Supporting information

S1 Table Additional themes and quotations. (DOCX)
