# Supplementary material for: A qualitative exploration of physical, mental and ocular fatigue in patients with primary Sjögren's Syndrome
Source: PLoS One. 2017 Oct 31;12(10):e0187272. doi: 10.1371/journal.pone.0187272 (PMC5663496; doi:10.1371/journal.pone.0187272)
Supplement: S1 Table — (DOCX) [file pone.0187272.s001.docx]

| **Theme** | **Quotations** |
| --- | --- |
| **Ocular dryness** | **My eyes I know are dry and I’ve always had dry eyes but I don’t really see anyone regularly about that and that worries me because I think are my eyes being damaged by the dryness, should there be somebody that I see on a regular you know six monthly basis to keep an eye on that so to speak.**  **So, you know, you still wouldn’t – blurry, blurry eyes, itchy eyes. I couldn’t see. You just don’t, well I didn’t [yeah], I didn’t.**  **my eyes seemed to get dry and irritated but seemed to be soothed by Hypromellose and no one seemed particularly concerned**  **But I did talk to the optician about it about two years ago and they suggested these sort of drops. But they're quite expensive to buy and I was managing, so I carried on with the cheap stuff, which probably in hindsight wasn't the best thing to do. But I really [05:16] my eyes got a lot worse.**  **Well my eyes were still dry and I used eye drops but I was a dental hygienist and worked under lights all the time and fairly [1:06] looking closely, I thought maybe that exasperated the dryness.**  **Some days I don't need many drops and another day I seem to be putting them in a lot. And what I do wear is I got them to make me up a pair of just plain glass, non-prescription, just plain glass, light-sensitive glasses that I wear when I'm out and about just to keep the air off. And that helps. And if I go into anywhere where it's central heated, if I go to the cinema or anything, I put them on. And it just stops the air - it keeps the moisture onto the eyeball.**  **If at home in the evening, if we have the gas fire on, which can be quite drying, then I have them on if I'm not - I'm old fashioned and I sit here doing knitting in the evening, so I have my reading glasses on then. But I generally have got some form of glasses on of an evening if I'm sat by the fire.**  **The only thing I had - for my eyes I have drops, I meant to bring them down. I have Refresh for when I'm driving for immediate relief and then I have - I haven't got one here. One which is more of an oily-based - hang on, I've got one. Oh, Systane. But, of course, they're quite oily so I can't use those if I'm going to read or do anything looking.**  **They look terrible most of the time, they’ve gone, I get a darkness round my eyes and the redness, but they don’t feel, it depends what I’m doing, at the moment they’re not very good because I’m trying to decorate my daughter’s house so of course, sanding things, although I wear goggles, just in the air, it makes your throat and your eyes a lot worse, but it’s nothing painful, so it’s not comfortable but it’s not painful.**  **I had dry eyes and they’d flare up and I’d get redness occasionally, and of course really I wasn’t lubricating them, do you know what I mean? It was just a simple thing really.**  **I know that a lot of people who have Sjögren’s have really really dry eyes and it must be really debilitating. but my eyes were tested last week and they were red, really quite dry but that really doesn’t bother me, but it’s the muscles with me, that’s my big thing.**  **Well, with my eyes, before I go to bed I tend to put some [19:08] eye gel in my eyes, and if I wake in the night to go to the toilet [19:15] I’ll put the same drops in, because my eyes are really…you can’t open them, they’re that dry.**  **things like in the car, if my husband puts the fan on in the car to clear the windscreen or something, that irritates, so any kind of like wind movement irritates them and things like that. And sometimes they do feel quite sort of dry and slightly itchy, but I wouldn’t say it was a major thing. It’s not a major thing that gets me down or anything like that.** |
| **Eye pain** | **I think the eye was going to go sore anyway and I think whatever I put in at that stage it was flaring up**  **Yeah and sometimes when they’re really, really dry they look almost like grains of sand, there’s like a little build up gets stuck round my iris and they’re just, that’s just excruciating the pain from that.**  **At that point in seemed to get worse, looking back, winter months and it was better in the summer. But I think that's just - I don't know whether that's true or not; it's just what I can remember. But I seemed to get conjunctivitis more frequently than I thought I should.**  **And sometimes it does feel as though I’ve got red hot pokers sticking in my eyes.**  **And they got more and more painful as time went by, and obviously they got [04:51 – gluey], they got drier and drier.**  **They were really, really sore, I was blinking a lot because I was trying to you know you could just feel that your eyes were dry. And at the end of the day particularly they felt very gritty as though something was in them you know you get something, the wind blows a particle of dust or something in, they were feeling like that. And I was having to sort of blink a lot to try and sort of you know help them really. And my eyes felt tired as well.**  **It’s definitely worse in the winter because like you say you tend to be shut up more and you’ve got the heating on and what have you. But I also find it uncomfortable if I’m out and it’s a windy day and you’re walking you know most people’s eyes they’ll water won’t they when the wind, mine don’t and they get so sore. It’s like chopping onions they’re, you know I don’t chop onions any more because I just, it’s just too painful because they don’t water as I say like a normal person for want of a better phrase as you’re chopping onions your eyes might water but mine don’t.**  **And it’s just one of things you have to get on with it really, the only time it really irritates me is if I think say we’re going out of an evening and my eyes have obviously been open all day and they get really sore then I do sort of make, I sort of have to think to myself around tea time I must just sit and shut them for a bit just to sort of help ease them.** |
| **Eye focus: Reading, computer** | **I’ve noticed myself when I’m trying to read small print, I’m having to sort of really concentrate on trying to focus and move it about to read it, whereas before I never used to have an issue, but I mean that could be my age more than anything**  **Now I can't because either I'm tired or my eyes can't cope, because by the evening the eye that's the worst gets really gritty, however many drops I put in it. So that's difficult because I'm not a morning person. [laughs] And my evenings are being impeded. I used to do a lot on the computer in the evening and that's not good either. So it's tricky**  **And watching television or going to the cinema when it's like that is very uncomfortable. I went to the theatre at the weekend and there were bright lights there**  **I mean, going on the computer’s bad. But, anyway, I tend to keep it to a minimum, which is difficult nowadays, because everything’s done by email and this and that. And, you know, they say, ‘Well, you can make the appointment by email,’ and I think, ‘Well, that’s the last thing I want to do, because I’ve got to [17:31 – fuss] with the computer.’ So things like that, I have discovered - again, by having a letter from one of my many consultants - that there’s a free talking book service, which is really good. I mean, all these things are worth people knowing, because I can, you know, they send me books, which I can listen to, so I can keep my eyes shut [okay]. Well, I used to like reading. That was a real loss. I used to sit in bed and read and I couldn’t anymore because it hurt …** |
| **Ocular irritation and light sensitivity** | **They are very effective to lubricate the eye but I need something that stops - I don't know how to help the worst eye to stop it getting - it's fine in the morning, although it's starting to get a bit irritated now. It's probably because I've done a little bit of a short drive in the sun, even with sunglasses on. Something I could use to calm that niggling feeling that there's a lump of grit in it. They're not itchy. I don't feel the need to scratch them but I'm aware that - the thing that most concerns - I think it's brought on by the light and I don't understand why that should be. Why should the light make them worse if my eyes are functioning all right and it's purely the tears? Is there more in the eye that's going on?**  **No, I don't think the medicines are making it more sensitive to light. I think the condition's made it more sensitive to light. They became really sensitive to light the last time I had conjunctivitis and remained so. Probably since I had that really severe inflammation, scleritis they called it. But probably since then I've noticed the light sensitivity. But whether that's caused something, I don't know**  **They're not getting gritty. They're not getting infected but it's become more light sensitive. So I've gone from one symptom to another. And I know that by halfway through the afternoon, early evening the bad eye will start to feel gritty. And watching television or going to the cinema when it's like that is very uncomfortable. I went to the theatre at the weekend and there were bright lights there**  **because I need tinted glasses all the time. I just worked that one out for myself. Two weeks ago I realised it wasn't any good trying to wear my glasses in the house even because of the light**  **Well, I've always worn sunglasses ever since I've been an adult. I've always needed to wear sunglasses and sunhats and things. I realised my eyes screwed up a lot in the sun. And I've always worn them for driving. But I've never been worried about walking around in the sun. My eyes have coped. But only in the last two months I've realised their light sensitive and it really shocked me the first time I was driving at night when it started and the headlights were just dazzling me. It was because my eyes were inflamed at the time as well. So I didn't realise that would happen too and that's got an awful lot worse**  **The light sensitivity is worse than anything else, to be quite honest, because it's quite tricky. I've recently had eye tests and sight but I've got to go back and talk to them about getting some prescription dark glasses, obviously. Ones I can wear all the time. But I don't normally wear glasses all the time but obviously I need to now. So I might as well have the prescription ones and I need to have the dark ones that don't go completely clear.**  **Yeah, that is the worst thing, is the air conditioning. So especially in somewhere like a theatre or cinema, that's bad. So I always have my ordinary glasses and I've had them, as I say, light - what do you call it? Light sensitive. Reactolite, aren't they? So they darken, so that's good. They don't look very elegant but they're okay**  **Yeah again it’s like I’ve said they, it depends what sort of atmosphere I’m in as well, if I’m in a dry, we work, we’ve got a carpenters and joiners business so I work there, I do the books and the payroll but I‘m in the office and there’s obviously dust in the atmosphere, it’s a hazard of the job. So they’re worse at work but then for example when I’ve been on holiday and it’s been hot the humidity it’s been brilliant, I’ve hardly put any drops in at all. I’ve noticed that for about the last seven years.**  **It isn’t because again you see I’m very, very lucky to be in the position that at least because it’s our business we’re in control of what’s in there. So I do the lighting that’s in my office suits me you know I’m extremely lucky to be in that position. But if I say sat here all day it would horrendous.**  **It’s like in the summer time when it obviously you know fingers crossed we get a lot of sunshine I’ve got those reactor light glasses as well because I tend to find a lot of sunlight you know my eyes get sore as well.**  **The light sensitivity is worse than anything else, to be quite honest, because it's quite tricky. I've recently had eye tests and sight but I've got to go back and talk to them about getting some prescription dark glasses, obviously. Ones I can wear all the time.**  **And over the last four weeks I've become extremely light sensitive.** |
| **Gritty and tiredness** | **Now I can't because either I'm tired or my eyes can't cope, because by the evening the eye that's the worst gets really gritty, however many drops I put in it.**  **They're not getting gritty. They're not getting infected but it's become more light sensitive. So I've gone from one symptom to another. And I know that by halfway through the afternoon, early evening the bad eye will start to feel gritty.**  **But interestingly enough, I've always had to wash my eyes in the morning from gritty, yucky stuff.** |
| **Fatigue (circadian)** | **And if I really, really get fatigue in the afternoon, I will just lie down, even if it’s on the sofa, just lie down, and don’t speak to anybody, and just, If I fall asleep, I fall asleep, and that’s it [yeah]. Participant 1.**  **Now I can't because either I'm tired or my eyes can't cope, because by the evening the eye that's the worst gets really gritty, however many drops I put in it. So that's difficult because I'm not a morning person. [laughs] And my evenings are being impeded. I used to do a lot on the computer in the evening and that's not good either. So it's tricky.**  **Yeah, but it doesn’t, when I say it doesn’t stop me doing anything, sometimes it used to, just for a little while, you know, like, it might stop me if you wanted to do something and you think, ‘Oh, I can’t do that, I’m too tired.’ But you get used to it and you just think, ‘Oh, well, I could do it next week,’ you know, just go for that week and miss it and then try again another day, and just try, even if you think it’s too difficult. Like, walking upstairs, for instance, I know I can walk up one flight and I can – it might be a difficult – but, you know, you need to do it. But once I get to two I’m, like, phew, phew, out of puff and [yeah], you know, but you just have to keep trying, that’s all [yeah, yeah], and you sort yourself out. But I try not to sit there and be a person who is like, ‘Oh, I don’t feel well today. Oh, I’ve got this today, I’ve got that today.’ And I see some of these people and I think, ‘For goodness, get off your backside and go for a walk in the fresh air,’ [yeah], and it’ll, sometimes that does more help, even if it’s just a little tiny bit down the road and back again, just outside the front door, it does help.** |
| **Stress and fatigue** | **I think possibly if I am slightly a little bit stressed or tired or whatever I know my feet tingle more, definitely. But then again, I suppose that's not my eyes or my mouth but I think that's part of your nervous system, isn't it? But I don't tend to get stressed nowadays. I just don't put myself in situations. I think it does play a role, definitely. And if you're low you're just generally much more susceptible to things. And it is an area that we're interested in and we want to try and find out whether [33:48 - doorbell rings] ... we're interested in whether stress management would help with the symptoms, both from a personality point of view, which you highlighted before, but for when people go through very stressful times in their life that either through psychological processes or through the immune system things tend to get worse or the experience of the symptoms gets worse and perhaps a psychological intervention to help them cope or to make sure that when stress happens in the future that they've already got the tools to cope better might help to alleviate some of the symptoms.**  **Yeah that’s what I need to think about yeah because that’s a bit of a worry for me but yeah and the other things I was talking about in my email to yourself wondering whether stress is a trigger for this type of illness. It’s like I had, like I say I had quite a, my dad was alcoholic so it was quite a stressful upbringing and of course my flight or fight reflex is very strong and there’s a lot, I imagine there’s lots of Cortisol or whatever, Adrenalin rushing around my body and has done for many years and I just wonder if that’s something that can trigger something like this looking back to how it could have been caused. And they don’t seem to know unless you know anything more but they don’t seem to know how this actually started in the first place do they really.**  **I don't know why, which is why I do pace myself a bit. I am conscious that I do need to be a bit aware of not overdoing it too much. So yes, I suppose I do - stress isn't good for me, I know that. So I'm lucky that I'm not in a particularly stressful job and I'm lucky that everything, touch wood, is not stressful at home and so forth.**  **IV: I know but you did...And it wasn’t until I came before that I realised all the different little things, and I thought, ‘Oh, I forgot about that. I’ve forgotten about that.’ I’d forgotten about my weight loss. I’d forgotten about all this [06:52] but always, stress, stress, stress. That’s what I always remember about that. They always say it’s stress.**  **I: But were you stressed?**  **IV: Well I was because I was feeling so unwell [okay, okay], [07:04 – but/when] everything else was just normal in life. It was just all, just normal, you know, your husband, your children, and that was it.**  **There is. Sometimes I’m embarrassed. I’m embarrassed because I can’t do as much as I want to do or used to be able to do.**  **Well, I was seeing the previous lady GP that I always used to see on the run up to Christmas because I got an acute bout of conjunctivitis and I got an acute bout of backache and then I collapsed and broke into tears because I was stressed because I was coping with my elderly parents and my own offspring and grandchildren were staying and it was all just chaotic. And so she and I rather put it down to stress and slight depression, but my eyes were bad as well.** |
| **Fatigue** | **And I was having to sort of blink a lot to try and sort of you know help them really. And my eyes felt tired as well. Participant 19**  **I also never know, I mean, they talk about the fatigue, and certainly by the evening, I can’t do anything, apart from not being able to see. But, again, I think, ‘How much is old age? How much is the Sjögren's?’ I think [18:51 - for] some people the fatigue is worse than mine. And it does tend to suddenly strike. I have noticed that, you know. I feel all right and then suddenly I think, ‘Oh, I’ve got to stop.’ But it’s funny that, isn’t it? It’s not as though you gradually get tireder and tireder [laughs]. It’s as though suddenly you just run out of just energy at all levels. It’s the emotional and mental and physical energy goes.**  **people can kind of, you sort of say well my eyes, they’ll say to me, oh you look tired, I’ll say I’m not particularly tired my eyes are sore and it’s oh right and they kind of get that.**  **Yeah I mean certainly like I said after being at work all day I couldn’t say come home and start reading or I’d come and I’d sort of just sit with my eyes closed for half an hour so just to try and give them some form of comfort really.**  **I can't forget, you see, because I do forget. Am more forget - I'm 63 now - so I have noticed I get tired a lot quicker in conversation. I'm tired now, and sometimes I'm tired and as soon as I get…it's the fatigue of the illness. I battle fatigue every day, but I don’t let anybody see it, but the fatigue is horrendous. Sometimes I could fall to sleep standing up. So yes, there we go.**  **And I think I disguise it quite well, to be perfectly truthful. People only see what I want them to see. They don’t see me going back to bed in the morning, they don’t see me trying to get out of bed at eight and can't get up, you know? They don’t see me falling to sleep and I cover everything up. So probably…that is quite bad, the fatigue, really dreadful, the fatigue. Yes, very tiring. This will whack me out now, this interview. Yes. But regardless of whether I'll be whacked out or not, I'll still get up tomorrow, shower, put my makeup on, get dressed, regardless of how weak I am, I'll be out there, nattering.**    **[So has the Sjögren’s affected your ability to be able to work?] I think it's going to, yes. I certainly couldn't do it for long hours and I'm not sure what it's going to be like if I work a long day. I think it is going to, yes. Luckily I'm only working part time now and I don't need to work full time. And it's certainly made me - I've been getting more tired over the last year or two, but at least the last year, and I just put it down to a very stressful year, my age and everything. But I understand now that you can get increased fatigue with this problem.**  **Only if I have a short sleep, very short sleep or I have a really disrupted night but I think that’s pretty normal, I don’t think it’s anything more so with Sjogrens really but I’m getting older you see. Participant 16.**  **Like I say I don’t suddenly bursting full of energy because I never do but I come out of that horrible can’t get off the couch because I could be on the couch for two days just have to sleep and rest and just can’t do anything.**  **I don’t, the only pattern I notice is like on the weekend I find I can sleep a lot over the weekend which isn’t particularly good with a family because obviously they need to get out and about and do things but I find I can sleep a lot over the weekend, it seems like I’m trying to catch up. And then I’ll be reasonably active, you know I can do what I need to do until about Thursday and then I start going downhill about Thursday so you know my daughter goes swimming Thursday evening and so after school I pick her up, come home, get the tea on and then it’s going out to swimming and that feels like climbing a mountain some Thursdays because it’s the week and I’ve had, I’ve sort of depleted my energy by then so I do see a bit of a pattern there [yeah]. My weekend is spent sort off catching up you know. Yeah I sort of think, oh my husband can share the load a bit now and I, you know because he’s at home then, I tend to sort of you know not really I don’t know I feel like I should be doing more really over the weekend, I should be doing more with the kids, I should be, but I just can’t really you have to sort of give in to it which is hard. Because you don’t want to but you just have to think well I just need to rest now you know so you just have to give in to it really. It’s definitely pacing and exercise is the things that do help so if I walk the dog most days and try not to pick too much into my week then that makes it manageable you know so yeah. The other thing looking at my email the other thing I mentioned was the chemical load whether Sjogrens was triggered by the chemicals and heavy metals that I worked with for a period of time, is there any research on that that you know of?**  **Yeah at least you feel like you’re trying to do something I mean to me it makes sense you are what you eat I think and you know if you eat protein that gives you energy then you need to give yourself protein, what I don’t understand is the mechanism that makes you tired whether you can overcome it by diet. But to me it makes sense that it should help but I don’t really know what causes the tiredness, I don’t really know the mechanism and I probably wouldn’t understand it because [24:22 – inaudible] I’m sure it’s very complicated.**  **I do I get very tense in my shoulders I tense up my muscles quite a lot and also I get tired because I’m working harder mentally and physically I’m tensing up so I think I get tireder as well so yeah I think it exacerbates things if anything [yeah] I try to keep that down to a minimum, I try not to, not to build too many things into my day and you know try and do things that are relaxing like I’ll take the dog for a walk and I do my art therapy, not art therapy but art which is like therapy for me [yeah] you know but yeah.**  **Well I just find every now and then it happened on a few occasions in the past and that’s why I’m linking it up to low [20:21 – inaudible] and I’ve gone to the doctor and said, look I’m just feeling dreadful you know I feel like I’m walking through treacle, everything I do even up the simplest unloading the dishwasher can feel like climbing a mountain it’s just ridiculous minor exertions can feel huge and I feel breathless. And she checked my bloods and the [20:44 – inaudible] level could be something, I think the range of [inaudible] is something like, it’s something ridiculous like 18 to 335 or something and I’ve come out at 20 and they’ve said, well you’re within range.**  **It’s quite hard work for people that haven’t got much energy and are a bit low it’s quite hard to try and get that hep you know it’s quite an effort I think sometimes you know there’s a lot going on in your body that you don’t really understand.**  **Yeah no it is it’s very difficult some days and sometimes, I mean I try and get out and about and do as much as I can and like today I’ve got two social things today to do which is great and part of me thinks should I go because I’m feeling so rough and I think no I’m going to go and I’m going to enjoy it and I’m going to have a nice time. And you sort of try and push it to the back of your mind, you have to push the pain and the tiredness say for that hour or two and then it comes back, you know you just try and ignore the pain I think that’s what people, you might find people might say that to you as well you try and ignore the pain and pretend it’s not there because if you dwell on it in some ways it’s quite depressing, it can make you quite nervous do you know what I mean so you have to sort of try and keep yourself above it in a way.**  **Fatigue is very bad I’m just finding I’m on the couch sleeping regularly, I had a couple of days last week and I’m suspecting my iron levels might be low I’ve not had a chance to get to the GP but I’m taking some iron tablets at the moment to try and boost that but generally I suffer from tiredness anyway particularly low at the minute. I get lots and lots of pain, aches and pains and fizziness, I get fizziness around my mouth, I get fizziness in my arms, I get foggy thinking so I feel like I have a conversation with someone and then I can’t remember what we’ve spoken about particularly I’ll forget the details. What else do I get, I get dry mouth like I say, dry eyes, tummy problems which I think are probably related as well and memory, like I say short term memory’s not very good either so there’s quite a few things really.**  **It is because you feel like you’re working hard to focus on the conversation and I think some of it is you’re distracted by the tiredness and the pain, you know I’ve got constant pain so that’s very distracting anyway. But some of it is just fogginess in my head and it is difficult because it’s got worse over the years, I was pretty sharp and very good at my job when I was working, very sort of switched on and I think that’s got worse over the years and the thought of doing something like I used to do now I just don’t know if I could do it really, I couldn’t take on a big mental challenge like I used to you know so that’s yeah that’s tricky really.**  **No, I don't. I'm lucky, I think. I know I've got the feet thing but I don't get the fatigue thing, which I think is a miracle considering. [laughs]**  **. And energy wise I’m thinking I can use food to give me more energy because of my tiredness so she’s [nutritionist] tried to do things like make sure I have enough protein over the day, make sure I stay hydrated over the day, she’s given me a plan to work to that might boost for my energy levels in some ways because that’s a big issue with me tiredness [okay].**  **Oh no, I'm retired. No, I'm retired. And I do have a busy day out or something like that and have a quiet day the next. I'm 70. I can't do the things that I rushed round and did when I was 40. I don't think anything about it. I spent four hours in the garden digging up some beds that had not been touched the other day. I've got plenty of energy, get up and go.**  **But yes, in terms of the fatigue I've got less energy than I used to have but I think that's probably an age thing. I'm more tired than perhaps I would be otherwise used to be because I used to be able to do the decorating and gardening and I'd just keep going and it wasn't an issue, whereas now I certainly wouldn't - I'd be able to do an hour maybe and then I'd need to have a break. But then I think my husband's the same. He's not got any issues but he's older and so consequently you pace yourself a bit more. So I don't think it's to do with the Sjögren’s.**  **But I look back and I used to be absolutely exhausted. And now, I mean, I need my sleep. I need a good eight, nine hours’ sleep and if I don’t get that, and my body tells me, but now I’m retired I can do that and I can do things at my own pace. So at the moment I really don’t [18:10 say I can feel] I mean, I’ll come out when we’ve been out for a day or something and we’ll come back and I do feel tired, but not the kind of exhaustion that I used to feel when I was younger.**  **I feel very very tired, at night I can’t sleep very well. I feel tried but I can’t sleep. My head, the back of my head is very very painful, on the left had side. It is really painful so I take paracetamol. I feel tired, I feel very very irritable at the moment. I was never, like that before, I’ve become very very bad tempered. I keep on having arguments with my husband, I’m very impatient, and I don’t know if this is due to my illness, I know I wasn’t like this before, I get anxious. If I want to do something, I get very very impatient, and I want to do it in advance. And sometimes I can’t think, and I can’t concentrate. Very difficult to concentrate. If we want to go somewhere, normally we go out dancing, and it’s hard to concentrate on the dancing. I read, and I have a lot of problems lately, I find that I can’t read very well. I can’t seem to see the writing, and my eye sight isn’t very good now. I have to write quite big, with my writing. When I’m reading my eyes do hurt me, it takes me a long time to read because I can’t see very well and it’s hurting, and I can’t ready very well, I get tired. I can’t watch the TV too long, I have to have a rest, normally I can’t watch the TV too long, I have to be in bed to watch it and then I fall asleep.**  **I think it would be good to have somebody to talk to. I think the counselling is useful, my Gp is very very busy anyway, and sometimes it helps to express yourself.**  **Yep, but I mean a lot of the adaptions have come on you know, I get up early in the morning you know, and I try to go to bed early of a night time, to try and help as best as I can, the tiredness, I try and be sensible and plan my days so that you know, I’m not running round at 2 o’clock in the morning and then, absolutely exhausted before I even start in the morning, because I feel tired anyway when I wake up in the morning, so I try not to overload in that way.**  **I am still absolutely exhausted in the afternoons or and the early evening, you know, I’m never out of bed past 11 o’clock if I can help it, but if I make it to then then that’s a bonus because by then normally I’m absolutely bushed and literally struggling at that point then, I feel like my body just doesn’t want to move anymore.**  **It’s trying to stay, you’ve got to try and stay positive because otherwise, especially when you’re tired, you feel lower, I find you know, if I’m tired, if something bothers me, I’m more likely to feel, well I say cry, but I can’t cry but you know what I’m saying, but for like, breaking down and saying I can’t do it, when you’re tired, but you’ve got to try and stay positive and look at the bigger picture and, I’m alive and I’m well, well as well as, you know, it could be a lot worse.**  **But it was just - I felt as if I was wading through treacle.**  **And I said I'd been really, really tired and he put it down to, 'Everything that's been chucked at you and a little bit of age,' although 60 isn't anything. But now I realise that you can get fatigue with this.**  **I'm coping with it but I find it quite limiting. And I get tired, I just sit down and fall asleep. But that’s manageable really, you just accept these things.**  **For years I wouldn't have been able to paint a wall for example in my house, when I would have just done it automatically. But to actually pick up a paint brush or a roller and try and do it, I just wouldn't be able to do it, everything would just fatigue. And I have to pay somebody to come and do the garden because in a way I couldn't even pull to start the mower. And it hurts. The most I've managed to do over the years is to go on a knee level and to work with a hand trowel. I couldn't work with a fork or spade. But now I can't even work with a hand trowel.**  **Yeah, quite busy. Oh no, I'm not - I don't have days when I have to just sit down. I'm doing decorating and that now and we've got quite a fair sized garden, so we have a go at that and grow vegetables.**  **Sometimes it's just everything completely aches, the whole body seems to ache and particularly if you’ve done something more active in the day, or two days before. But for a while you can feel reasonably normal and then all of a sudden it's just like an overpowering shatterness, you just can't stay awake, I've sat down here and I've just slept for two or three hours or maybe an hour here and then an hour again in the evening and then gone to bed. But I always sleep a good night. I do [37:58] I would think sleep at night.**  **The fatigue, all of a sudden you can be out shopping and you think, ‘Oh, my God, I’ve gotta go and sit down.’ So, if I’m with somebody I’ll go and have a cup of coffee, or sit for an hour and have lunch or something. And if I’m at home I just lie down, I just lie down, and even if you just lie, if I could just lie for half an hour, it makes a difference. But then I’ll get up and try to work through it, but you can’t always do that, and sometimes just a little sleep or self-help is really good with Sjögren's, that’s my…that’s my opinion [yeah, yeah, yeah], you know, and I try to exercise with yoga, tai chi.**  **But concentration is much more difficult; it was difficult before but it’s even more difficult now. Yeah, staying focused, and short-term memory appalling now, appalling [yeah], yeah, that I’ve noticed, yes [yeah]. It absolutely does my head in [laughs], yeah. Also, because all of the three things together, the tiredness [08:34], the – I can’t get my words out now [laughs] – that kind of thing, the whole [08:40 - three] of those things, when you’re in a conversation, if it’s quite a fast conversation, I find that difficult to do now, whereas before I could just, you know, do all of that.**  **I was more tired. So I’d gone to the doctor’s and he tested me for diabetes and it was fine. So, I just thought, ‘Oh, it must be just I’m doing too much.**  **Some days I’m all right and I can keep going [yeah], but some days it’s just, you know, I’ve just got to get on there and get my head down.**  **I was suffering with tiredness, but I’ve got a son with ADHD and Asperger’s, so part of me sort of, gets used to being tired [laughs], so I sort of put a lot of it down to being just generally running round like a headless chicken, although it did feel, I did feel more [02.28] sort of thing.**  **Sometimes I do struggle word search and sometimes I can't identify things, but again, it isn't a problem because as long as I can get to the shops, get my food, I know what I'm eating, I can get on the bus, I can travel, I can get from A to B, I'm fine. So there we go. Sorry, I've forgotten what the question was?**  **And after about an hour I found, ‘What’s the matter with me? My eyes are really hurting and, you know, is it just because it’s in the evening and I’m tired?’ And that was the first I noticed, really.** |
| **Fatigue: work** | **No. Because of the trouble with my legs, so that I can’t stand or sit for too long, or if I do, once I then move [09:20] kicks in, and I have good days and bad days. I can’t see how I can do a job because you can’t have somebody like that working for you. So I do, kind of, some voluntary stuff, but I do find that some afternoons I’ve just got to go and lie down and I sleep, and then I wake up and I don’t feel horrible, I’m fine, and then I can cope with the rest of the day. So, again [laughs], you can’t do that in the workplace.”**  **“And then I suppose I took life changing…actually I realised I couldn't cope with what I'd been doing and the work that I'd been doing,”**  **And I was just too tired, my arms and everything would ache, I just couldn't hold my arms up like that to do that work all day. And then I had the cancer so I gave up work.**  **Definitely, definitely I think they should. I don’t know how they could do that but they definitely need to know more of the things that you can get, and more of your symptoms. That’s what I think, anyway. And I think if they knew more about Sjögren's and the symptoms and what can be caused by it, I think they would understand more, even if they just went to a one-day course. That’s what I think…….Just to know that this can be [yeah, yeah], ‘Sjögren's can cause this,’ and, ‘people feel like this, and people feel like,’ you know, whatever. But how could you do that to all the GPs in the country?** |
| **Eye fatigue** | **I just felt, ‘I’ve gotta stop. I’ve just gotta shut my eyes. They’re just hurting.’ So that has been a good find.**  **Now I can't because either I'm tired or my eyes can't cope, because by the evening the eye that's the worst gets really gritty, however many drops I put in it. So that's difficult because I'm not a morning person. [laughs] And my evenings are being impeded. I used to do a lot on the computer in the evening and that's not good either. So it's tricky.**  **But I’ve learnt to somehow…It took a long time to live with it, but other times they’re just hurting so much that, really, all I can do is, make sure I’ve got loads of drops in and shut them for a while. And I find by the time the evening comes, they’re usually so sore that they’re pretty useless, really**  **Not that I’ve noticed, not that I’ve noticed, I think, obviously in the morning they feel worse because you’ve been asleep, so my eyes are gunged up and sore and what have you.**  **No, I don't think the medicines are making it more sensitive to light. I think the condition's made it more sensitive to light. They became really sensitive to light the last time I had conjunctivitis and remained so. Probably since I had that really severe inflammation, scleritis they called it. But probably since then I've noticed the light sensitivity. But whether that's caused something, I don't know.**  **They're not getting gritty. They're not getting infected but it's become more light sensitive. So I've gone from one symptom to another. And I know that by halfway through the afternoon, early evening the bad eye will start to feel gritty. And watching television or going to the cinema when it's like that is very uncomfortable. I went to the theatre at the weekend and there were bright lights there and [31:15].**  **I think it's brought on by the light and I don't understand why that should be. Why should the light make them worse if my eyes are functioning all right and it's purely the tears? Is there more in the eye that's going on?**  **I need to go to the optician and get some different glasses, because I need tinted glasses all the time. I just worked that one out for myself. Two weeks ago I realised it wasn't any good trying to wear my glasses in the house even because of the light.**  **I realised my eyes screwed up a lot in the sun. And I've always worn them for driving. But I've never been worried about walking around in the sun. My eyes have coped. But only in the last two months I've realised their light sensitive and it really shocked me the first time I was driving at night when it started and the headlights were just dazzling me. It was because my eyes were inflamed at the time as well. So I didn't realise that would happen too and that's got an awful lot worse.**  **My eyes, and I cannot read a thing of an evening, unless I’ve got a really bright light. I’ve got one, it’s, a white light I think it’s called, or something, and then you can read something.**  **I: Yeah, like one of those halogen type [yeah] things, yeah?**  **IV: Yeah, it’s white light.**  **So I always have my ordinary glasses and I've had them, as I say, light - what do you call it? Light sensitive. Reactolite, aren't they? So they darken, so that's good.**  **Well my eyes were still dry and I used eye drops but I was a dental hygienist and worked under lights all the time and fairly [1:06] looking closely, I thought maybe that exasperated the dryness. Participant 3.**  **Well I'd had a problem with dry eyes at least eight or nine years ago, maybe even ten years ago. And I just managed it with Hypromellose and I didn't really think anything of it. If I asked the doctor about it they said, 'Partly your age and partly if you spend time on the computer it's going to get worse.' And that was it, really, for a few years.**  **It [eye fatigue] can happen at any point because I feel tired a lot, you just, they just feel very heavy, very gritty and it’s just, you feel like they’re struggling to open, but I mean, with mine it’s more of a night time, I’ll just sleep but it can happen at any point. Participant 10.**  **My eyes, trying to keep my eyes open, just, you know, you just feel that exhausted, that you just, you can’t seem to get yourself the umph to even, you know, and you’re sort of going upstairs to go to bed, it’s just oh God, I’ve got to go up.**  **That I have dry eyes and dry mouth, and things like that. And she said oh it doesn’t matter, they did nothing actually. The first time I got referred I went to my doctor, I had dry eyes and I had an eye operation, I had my cataract done..**  **Even with the eye drops there’s no improvement. My eyes are terrible, even this morning, I still feel quite a lot of eye drops, then they tell me to put some eye drops in to my eye. Sometimes it’s very painful, and sometimes it scratchy as well.**  **It’s not some much that they [eyes] are tired, they are just very very dry. The itchiness can prevent me sleeping at nights. I usually get 4 or 5 hours, sometimes I don’t sleep at all. If I get itchiness I don’t sleep. It’s the itchiness that prevents me from sleeping. I take a medicines, it prevents me itching, what’s it called, antihistamine, it’s an antihistamine I take that helps me with the itching. I take 5 milligram or 10 milligram.**  **I wake up alright in the morning, I usually get up at 5.30-6 am, and then I walk a mile every morning. it helps my leg, and I think the walking has done me a lot of good. I started walking and I feel slightly better. And when I go for a walk I feel me relaxed actually.**  **It's very dry. It does become very sore and can be quite painful and very probably in the - well, even when I put drops in but blurry in the evening, so - I'm very light sensitive as well. I have to wear sunglasses even when it's not even that bright. And I stay out of the bright sun when on holiday.**  **Yes. So I don’t feel like I can read in an evening, for example, because they're just so sore and tired. I certainly don't drive any more in the evening because I don't feel confident that my eyes - that I'm seeing clearly.**  **Gradually my eyes get more sore as the day goes on. They're very sore in the morning. I put loads of eye drops in. And it depends whether there's central heating or whether there's a wind blowing and all of that sort of thing. For example, where I work I'm looking at a computer all day and I find that they get very, very tired and sore by the end of that. So, for example, then I'm unlikely to come home and start reading much because they're sore and tired. They do get a bit blurry, I think, in the dark in the evening. I don't know whether that's to do with that or what that is. But I don't wear glasses for reading.**  **You sort of think 9 o’clock I need to go to bed now and I don’t read as much as I used to which is a shame and it’s just due to because they’re sore, they get sore.** |
| **Interrupted sleep.** | **But because I’m drinking constantly I usually have to get up to go to the toilet in the night and depending on what time it is sometimes you’ve had several hours sleep you can’t get back to sleep. It’s like this morning I’ve been awake since 4.15 because I got up to go to the toilet and I was like really dry and you just can’t get back off, it’s a nightmare.**  **You feel like shattered, come 1 o’clock you’re just you know, that’s why I don’t go to bed late.[okay]. I mean one of the nights over the weekend it was 6.30 and I thought crikey, it feels almost like you’ve got a newborn baby, great you’ve slept all through the night, it’s unbelievable really you don’t very often have a full night’s sleep.**  **I do but I sometimes think a lot of that is the lack of sleep like I said it’s not for the want of trying and it’s like I said I do not go to bed late but I just you know I do sometimes, some days are worse than others with that but again I try not to sort of let it stop me doing things because I think if you do that you’ve lost the battle haven’t you, you give in and I’m not, that’s not me.** |
